# Acute Sarcopenia Secondary to Hospitalisation - An Emerging Condition Affecting Older Adults

**DOI:** 10.14336/AD.2017.0315

**Published:** 2018-02-01

**Authors:** Carly Welch, Zaki K. Hassan-Smith, Carolyn A. Greig, Janet M. Lord, Thomas A. Jackson

**Affiliations:** ^1^Institute of Inflammation and Ageing, College of Medical and Dental Sciences, University of Birmingham, Edgbaston, Birmingham B15 2TT, UK; ^2^Queen Elizabeth Hospital Birmingham, Edgbaston, Birmingham: B15 2WB, UK; ^3^Institute of Metabolism and Systems Research, College of Medical and Dental Sciences, University of Birmingham, Edgbaston, Birmingham B15 2TT, UK; ^4^Centre for Endocrinology, Diabetes and Metabolism, Birmingham Health Partners, University of Birmingham, Edgbaston, Birmingham B15 2TT, UK; ^5^School of Sport, Exercise & Rehabilitation Sciences, College of Medical and Dental Sciences, University of Birmingham, Edgbaston, Birmingham B15 2TT, UK; ^6^MRC Arthritis Research UK Centre for Musculoskeletal Ageing Research, University of Birmingham, Edgbaston, Birmingham B15 2TT, UK

**Keywords:** sarcopenia, disuse muscle atrophy, muscle wasting disorders, acute illness, hospitalisation

## Abstract

There has been increasing interest and research into sarcopenia in community-dwelling older adults since the European Working Group on Sarcopenia in Older People (EWGSOP) agreed a consensus definition in 2010. Sarcopenia has been defined as loss of muscle mass with loss of muscle function (strength or physical performance), with measurements two Standard Deviations (SDs) below the mean of a young reference population. This definition does not necessitate longitudinal measurements, or the absence of acute illness and diagnosis can be made from single measurements. We hypothesise that hospitalisation, due to a combination of acute inflammatory burden and muscle disuse, leads to an acute decline in muscle mass and function and may lead to some individuals meeting criteria for sarcopenia, acutely, based on the EWGSOP definition. This may be partially recoverable or may lead to increased risk of developing sarcopenia long-term. We have denoted the term “acute sarcopenia” to refer to acute loss of muscle mass and function associated with hospitalisation. This review discusses some of the current available research in this context and also identifies some of the knowledge gaps and potential areas for future research.

Sarcopenia refers to age-related loss of skeletal muscle mass and function; the term originates from Greek, meaning literally “loss of the flesh”. The European Working Group on Sarcopenia in Older People (EWGSOP) [1], International Working Group on Sarcopenia (IWGS) [2] and Asian Working Group for Sarcopenia (AWGS) [3] all define sarcopenia as loss of skeletal muscle mass and muscle function; either low muscle strength or low physical performance. Different cut-off values have been proposed dependent on the population, however, both the EWGSOP and AWGS have proposed cut-off points of measurements two Standard Deviations (SDs) below the mean from a reference population of healthy young people [1, 3]. The EWGSOP defines the presence of all three criteria (low skeletal muscle mass, low muscle strength and low physical performance) as severe sarcopenia [1]. It should not be viewed as inevitable that all people will meet criteria for sarcopenia diagnosis as they age. The presence of sarcopenia is associated with increased risk of potentially detrimental outcomes including limitations upon quality of life [4], increased risk of falls [5] and increased mortality [6].

The presence of sarcopenia has been demonstrated to relate more closely to adverse outcomes than reduced muscle mass alone [7]. The EWGSOP has defined the presence of low muscle mass alone as “pre-sarcopenia” [1]. Another term that has been proposed is “dynapenia”, which refers to the presence of reduced muscle strength regardless of muscle mass [8]. However, the association between muscle mass and muscle strength is non-linear [9]. Sarcopenia is an all-encompassing term denoting both loss of muscle mass and muscle function, which has clinical importance and is widely used [1].

Sarcopenia is distinct from but related to the diagnosis of frailty [1, 10]. This is the syndrome of multiple cumulative decline resulting in increased vulnerability to poor resolution of homeostasis after a stressor event [11]. Frailty is a measure of the observed heterogeneity among older people and increasing frailty is associated with risk of adverse outcomes such as new institutionalisation and mortality. In the hospital setting, frailty is also associated with an increased risk of adverse outcomes following hospitalisation or acute illness [12-14] and should be considered when drawing conclusions related to sarcopenia.

The EWGSOP refers to the principle that both primary and secondary forms of sarcopenia exist; primary sarcopenia has no other cause other than cumulative insults over time associated with ageing, whereas, secondary sarcopenia may be disease-related or nutrition-related [1]. The AWGS also proposes a dynamic approach to sarcopenia evaluation including measurement of changes of muscle mass and muscle function over time [3]. There has been significant research into the development of sarcopenia in community-dwelling older adults and associated outcomes, but there is a paucity of research relating to associations between sarcopenia and acute illness. We reason that hospitalisation for an acute illness or surgical procedure may be expected to precipitate the acute development of secondary sarcopenia due to an increased inflammatory burden in combination with muscle disuse [15]; we refer to this condition as “acute sarcopenia”.

The frequency of acute sarcopenia is currently unknown, but it is known that a higher proportion of individuals will meet criteria for sarcopenia at any one time in hospital compared to in the community. Acute sarcopenia is a significant problem and important area for future research. It is likely to be associated with increased financial cost secondary to increased length of stay, rehabilitation needs and increased social care needs [16, 17]. It is known that bed rest leads to disuse muscle atrophy in healthy adults [18] and that inflammation leads to a heightened catabolic state [15]. We propose that the term acute sarcopenia should be used as an overriding term applying to significant changes in both muscle mass and muscle function associated with increased inflammatory burden combined with muscle disuse. The aim of this review is to summarise existing observations and hypotheses relating to acute sarcopenia observed in older adults during hospitalisation and how this condition relates to chronic sarcopenia.

## Sarcopenia

### Chronic sarcopenia: prevalence, pathology and outcomes

The prevalence of sarcopenia amongst older community-dwelling adults, using EWGSOP criteria, has been variably reported in 15 studies in economically developed countries as between 1-29%, in a recent systematic review [19]. Prevalence of sarcopenia rises with increasing age [20] and an increased prevalence of 32.8% amongst nursing home residents has been reported [21]. The prevalence rate of sarcopenia amongst older hospital inpatients has been reported at 22.1-26.0% in four studies [22-25].

The precise mechanisms that lead to sarcopenia are only partially understood. However, it has been hypothesised to be related to an accumulation of multiple insults over time [1]. Reduced dietary intake of protein [26], reduced physical activity [27], vitamin D deficiency [28, 29], cumulative inflammatory insults [30, 31], oxidative stress [32] and resistance to anabolic stimuli [33] have all been postulated to contribute. Mitochondrial dysfunction [34] and muscle denervation [35] have been implicated. There are currently no recognised histopathological features that necessitate a diagnosis. However, reduced muscle fibre cross-sectional area, with a preference for Type 2a skeletal muscle fibres [36] and reduced satellite cell numbers [37] are associated with ageing and found in sarcopenia in humans. Increased numbers of senescent satellite cells associated with reduced muscle mitochondrial function have been reported in aged mice [38].

Although individual differences exist and in particular a high level of physical activity in adulthood can prevent sarcopenia [39], a decline in muscle mass with age is seen in a majority of people. Importantly, the presence of sarcopenia is associated with increased risk of adverse outcomes. A prospective longitudinal study involving older adults in Belgium demonstrated reduced quality of life associated with physical function amongst sarcopenic individuals, although they also had a higher rate of frailty [4]. The relative risk of falls with the presence of sarcopenia has been reported as 1.82 (95% CI 1.09-2.18) [5]. A cohort study of octogenarians conducted in Italy revealed excess mortality amongst sarcopenic individuals with a hazard ratio of 2.32 (95% CI 1.01-5.43), after controlling for confounders. Measurements of impairments of Activities of Daily Living (ADLs) and other markers of physical frailty were controlled for, but frailty itself was not measured [6]. Frailty and sarcopenia are related conditions, but should be considered as distinct.

### Chronic sarcopenia: Inflammatory and Endocrine influences

Inflammatory cytokines such as Interleukin 6 (IL-6) and Tumour Necrosis Factor Alpha (TNF-α) have been proposed to contribute to catabolism in skeletal muscle. Inflammatory cytokines activate Nuclear Factor Kappa-light-chain-enhancer of activated B cells (NFκB) and Forkhead box O (FoxO) transcription factors in muscle resulting in proteolysis via induction of atrogenes [40]. Raised levels of the pro-inflammatory cytokines, IL-6 and TNF-α have been observed in frail and sarcopenic vs. non-frail or non-sarcopenic cohorts [31, 40-47]. High Sensitivity C-Reactive Protein (hsCRP) is a non-specific but highly sensitive marker of inflammation. Increased hsCRP measurements have been demonstrated in frail individuals and in patients with reduced muscle function [46, 48-50]. The ageing group of a population based observational cohort study demonstrated that frail patients with raised hsCRP measurements were most likely to be admitted to hospital [51]. Elevated hsCRP levels are also associated with reductions in muscle strength [52, 53] and physical performance [52] although no consistent relationship with sarcopenia has been found [53].

Endocrine and inflammatory status interact to modulate muscle mass. Cortisol levels have been demonstrated to increase slightly with age, which in turn is associated with muscular weakness. A cohort study of adults aged 65 and older in the Netherlands demonstrated that increased salivary but not serum concentrations of cortisol were associated with reductions in grip strength [54]. 11β-hydroxysteroid dehydrogenase type 1 (11β-HSD1) regulates glucocorticoid exposure at the prereceptor level; the type 1 isoform acts as an oxoreductase converting cortisone to active cortisol in humans and can be induced by pro-inflammatory cytokines such as TNFα. Skeletal muscle expression of 11β-HSD1 has recently been demonstrated to be negatively associated with grip strength in both men and women and with total lean mass in men [55]. Transgenic animal models have demonstrated that inactivation of 11β-HSD1 is protective against skeletal muscle atrophy precipitated by the administration of exogenous glucocorticoids [56]. 11β-HSD1 has been demonstrated as a major regulator of intramyocellular protein metabolism, impacting upon myotube size in both animal and human models and expression of a number of genes involved in protein synthesis, growth factors and the ubiquitin proteasome system [57].

Additional endocrine regulation occurs through growth hormone (GH) which induces insulin-like growth factor 1 (IGF-1) production in the liver. Both insulin and IGF-1 receptors are upstream of phosphatidylinositol 3 kinase/ protein kinase B (PI3K/AKT) pathway activation and can trigger protein synthesis. The PI3K/AKT pathway stimulates protein synthesis via activation of glycogen synthase kinase 3 (GSK-3) and mechanistic target of rapamycin (mTOR) pathways and prevents protein degradation via the FoxO pathway [58].

Vitamin D has also been implicated in modulation of skeletal muscle mass. Muscle biopsies taken from patients with vitamin D deficiency have demonstrated atrophy of Type II muscle fibres [59]. Additionally, vitamin D correlates with physical performance measures, although this may be related to bone strengthening as well as muscle function [59]. Active vitamin D levels have also been demonstrated to correlate with muscle strength in healthy younger adults [60].

### Associations between chronic sarcopenia and hospitalisation

An increased risk of hospitalisation amongst individuals with sarcopenia compared to those without sarcopenia has been reported in a longitudinal study in the USA [28]. Similarly, the presence of frailty has been shown to increase the risk of hospitalisation [61]. It has been shown that hospitalisation itself is associated with functional decline. This is most marked in frail patients [12, 14]; frail patients admitted to hospital have longer lengths of stay and increased mortality [13]. Sarcopenia has also recently been demonstrated to be associated with increased lengths of stay, although, interestingly, this effect was more significant amongst younger individuals [62].

Few studies have assessed sarcopenia criteria during the acute phase of an illness. A prospective observational study involving 103 older adults admitted to geriatric medicine wards, who had evidence of or who were felt to be at risk of malnutrition, demonstrated a prevalence of sarcopenia of 21.4%. Notably, 22.3% of patients recruited to this study were unable to perform both the gait speed test and handgrip measurements due to acute illness. An association was seen between sarcopenia and increased mortality; further research is needed to assess the cause for this association [63].

The presence of sarcopenia is associated with increased costs and mortality in general, vascular and liver transplant surgery specialties [16]. Reduced muscle mass was independently associated with post-operative complication rates and mortality amongst patients aged 80 years and older undergoing emergency surgery in a Canadian study [64]. Similar findings have been found in studies involving older patients admitted for colorectal surgery [65-68]; sarcopenia is associated with adverse outcomes following colorectal surgery in patients aged 65 years or older, but not in their younger counterparts [66]. Following colorectal surgery, older adults experience increased rates of post-operative infections, higher frequency of need for rehabilitation and longer lengths of stay [66].

## Acute muscle wasting disorders

### Bed rest and disuse muscle atrophy

Loss of lean leg mass during periods of bed rest in otherwise healthy adults has been extensively reported [15, 18], muscular disuse alone thus contributes to loss of muscle mass secondary to hospitalisation. Crucially, the effect of bed rest is more marked in older adults; five days of bed rest resulted in loss of lean leg mass and strength in older but not younger adults in a recent study [69]. Interestingly, a study involving middle-aged adults demonstrated that 14 days of bed rest resulted in reduction of cross-sectional area of muscle fibres, with a preference for Type 2a fibres and reduced satellite cell content [70]. These changes are similar to those that have been described with sarcopenia [36, 37], suggesting that bed rest may lead to an acute acceleration of this effect.

In rodents, skeletal muscle unloading has been most commonly modelled using hind limb immobilisation. The molecular mechanisms that have been implicated in the development of disuse muscle atrophy are the Atrogin-1/ Muscle atrophy F-Box (MaFbx)/ Muscle ring finger 1 (MuRF1) pathway, the IGF-1-AKT-mTOR pathway and the Myostatin pathway [71]. MaFbx and MuRF1 mRNA levels rise in rodent models of immobilisation; this rise is associated with increases in proteolysis but not inhibition of protein synthesis [72]. The presence of IGF-1 has been demonstrated to prevent proteolysis [73] and the AKT-mTOR pathway is downregulated in rodent models of muscle atrophy [74]. Myostatin also inhibits protein synthesis via the AKT-mTOR pathway; myostatin (also referred to as GDF-8) knockout mice have experience dramatic muscle hypertrophy [75].

Immobilisation studies in humans have shown that the mechanisms involved include induction of the atrogenes MuRF1, Foxo3 and atrogin 1, with just 8 days of immobilisation leading to a 51% increase in MuRF1 protein [76].

### The acute illness effect

Hypercortisolaemia has been demonstrated to exacerbate loss of muscle mass and muscle strength associated with bed rest. A controlled study involving timed hydrocortisone administered to healthy young men over a 28 day period of bed rest demonstrated greater loss of lean leg mass compared to a bed rest only model [77]. Cortisol is a mediator of protein catabolism and serum levels rise significantly with acute illness and stressor events [78]. In addition, with age the serum levels of the androgen precursor dehydroepinadrosterone sulphate (DHEAS) decline, producing an increased cortisol: DHEAS ratio and a state of relative cortisol excess. Acute illness or stress such as a hip fracture will increase this imbalance further compromising muscle anabolism. Studies in elderly hip fracture patients have shown an increased cortisol: DHEAS ratio and this was associated with increased physical frailty up to 6 months after injury [79]. This suggests that changes in muscle mass and muscle function associated with hospitalisation are not related to bed rest alone and are compounded by the effects of acute illness; the cortisol: DHEAS is likely to be one of these mediators.

As described above the development of sarcopenia is positively related to oxidative stress [32] and inflammatory insults [30, 31]. Systemic markers of inflammation rise acutely with acute illness and stressor events and may act to potentiate acute loss of muscle mass and function via this mechanism [80]. This could be via a direct effect on atrogene levels, or induction of 11βHSD1 [57]. MaFbx and MuRF1 upregulation have been implicated in muscle atrophy; rodent models of sepsis induced by caecal ligation and puncture have been demonstrated to lead to increases of MaFbx and MuRF1 mRNA levels [72]. These two atrogenes are ubiquitin ligases involved in the ubiquitin proteasome system (UPS); multiple inflammatory disease states have been demonstrated to be associated with increased levels of MAFbx and MuRF1 as well as components of the UPS. Further research in human studies is warranted to better evaluate these pathways in relation to inflammation [81].

### Intensive Care Unit-Acquired Weakness (ICU-AW) - part of a spectrum?

Intensive Care Unit-Acquired Weakness (ICU-AW) is a recognised complication following admission to the Intensive Care Unit (ICU). ICU-AW is an all-encompassing term including critical illness myopathy (CIM), critical illness polyneuropathy (CIP) and critical illness neuromyopathy (CINM) [82]. Prolonged lengths of stay in the ICU are associated with increased loss of quadriceps muscle mass, suggesting that early intervention in this group of patients might help to conserve muscle mass [83].

The presences of sepsis or Systemic Inflammatory Response Syndrome (SIRS) criteria are known risk factors of ICU-AW. Local inflammation is found in ICU-AW, although specificity is unknown. Fourteen studies examining the presence of inflammatory cells within human muscle biopsies demonstrated a positive finding in 25% of cases (95% CI 18-34) [82]. One study assessed for the presence of cytokines in muscle biopsies taken from 30 patients with ICU-AW; muscle biopsies from two ICU patients without ICU-AW were used as controls. The presence of TNFα (90% vs. 0%) and IL-10 (96% vs. 0%) was found to be significantly higher in biopsies taken from patients with ICU-AW [84]. Multi-organ failure is also associated with accelerated muscle wasting, when compare to single organ failure [85].

**Figure 1.**
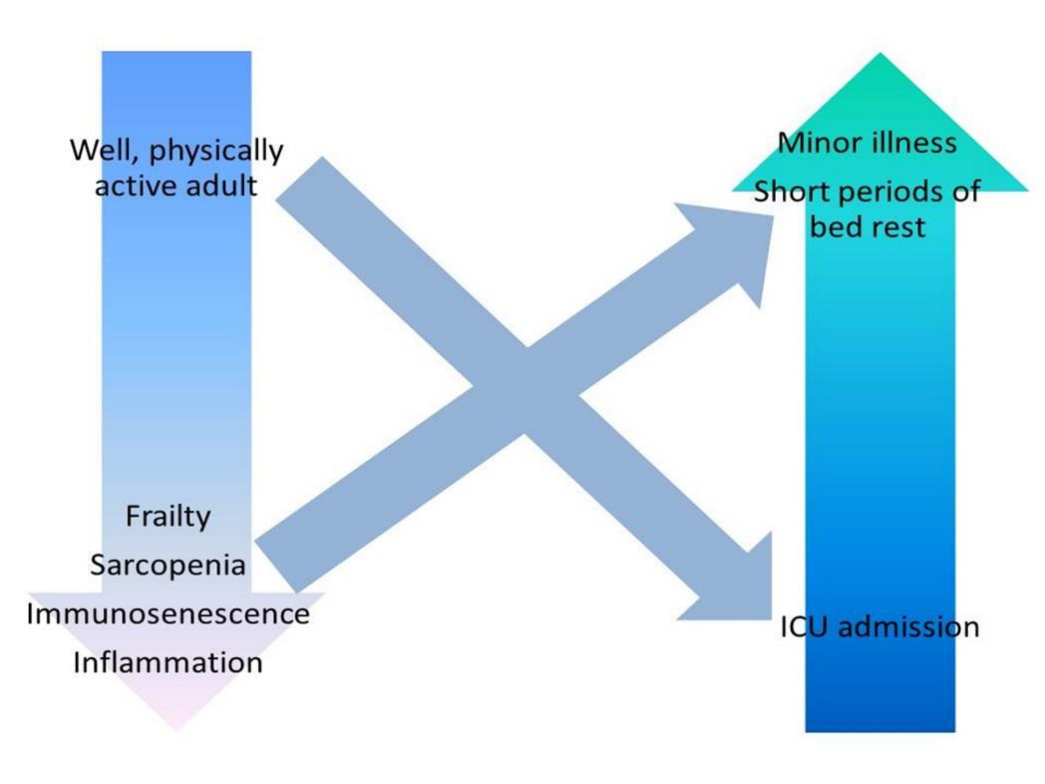
Acute sarcopenia as part of a spectrum of acute muscle wasting disorders This schematic demonstrates the relationship between underlying predisposing factors and precipitating factors resulting in acute sarcopenia or acute muscle wasting. Physiologically fit adults who are immunocompetent may experience significant muscle wasting in the context of a critical illness necessitating admission to the intensive care unit. Conversely, frail older adults with immunosenescence may develop acute sarcopenia following a seemingly minor physiological stressor event such as a mild infection or short periods of bed rest.

ICU-AW affects comparatively younger individuals than age-related sarcopenia, even though these patients may not have been exposed to multiple previous episodes of oxidative stress or inflammatory insults. However, age is a risk factor for ICU-AW, a USA study reported a mean age of 59.73 years in those with ICU-AW compared to 49.98 in those without [86]. Frail older adults are known to be at an increased risk of adverse outcomes following ICU admission and are often prevented from ICU admission to avoid unnecessary burden and harm [87]. It may be that ICU-AW would be catastrophic to this group of patients, presuming they survived the admission itself. ICU-AW may be related to the acute loss of muscle mass and muscle function experienced by older adults following hospitalisation or may be part of a spectrum of the same condition [88]. Figure 1 depicts our proposed model of the relationship between vulnerability and acute loss of muscle mass and function.

## Acute sarcopenia

### Acute sarcopenia: a proposed concept

Current available research suggests that hospital admission is likely to have a negative impact upon muscle mass and muscle function [89]. This leads to overall worse outcomes, in terms of increased lengths of hospital stay, increased rehabilitation needs and increased discharge of patients to institutional care [16, 17]. We hypothesise that this is due to a combination of muscle disuse [18], endocrine dysregulation [90] and acute inflammatory burden [82]; these effects are likely to be compounded by age and previous cumulative insult [69]. In order to meet criteria for acute sarcopenia, rapid reductions in muscle mass and muscle function should be demonstrated; this requires that measurements of muscle mass and muscle function are available either pre-illness or during early stages of an illness.

We define acute sarcopenia as changes in muscle mass and muscle function within 28 days of a significant physiological stressor event, such as an acute illness, surgery, trauma or burns [15, 90], sufficient to newly meet criteria for sarcopenia using previously defined cut-off points [1-3]. However, changes in muscle mass and function that are significant but that do not meet criteria for sarcopenia may have negative long-term consequences; further research is necessary to evaluate this [15]. Additionally, there may be an acute-on-chronic phenotype in some individuals; older adults who have sarcopenia may develop sarcopenia following an acute illness [1].

As a specific example, sepsis is a life-threatening condition characterised by the presence of infection associated with organ dysfunction. Survivors of severe sepsis are at an increased risk of developing new cognitive impairment or functional disability long-term [91]. Sepsis is associated with an acute upsurge in inflammatory markers and acute decline in muscle function [92]; this may impact upon the individual long-term. However, surgery, trauma and burns [93] are also associated with endocrine dysregulation and acute inflammatory burden and may similarly impact upon muscle mass and muscle function. Conversely, a frail older adult may be at increased risk of loss of muscle mass or function following a relatively minor illness due to a reduced immune response (immunosenescence) (see Fig. 1); immunosenescence affects an individual’s ability to overcome an infection or injury and may lead to sustained effects [94]. Altered neutrophil migratory dynamics for example occurring as part of ageing, lead to increased tissue damage from excess release of neutrophil elastase and resultant inflammation [95]. This in turn will contribute to muscle loss and frailty.

**Figure 2.**
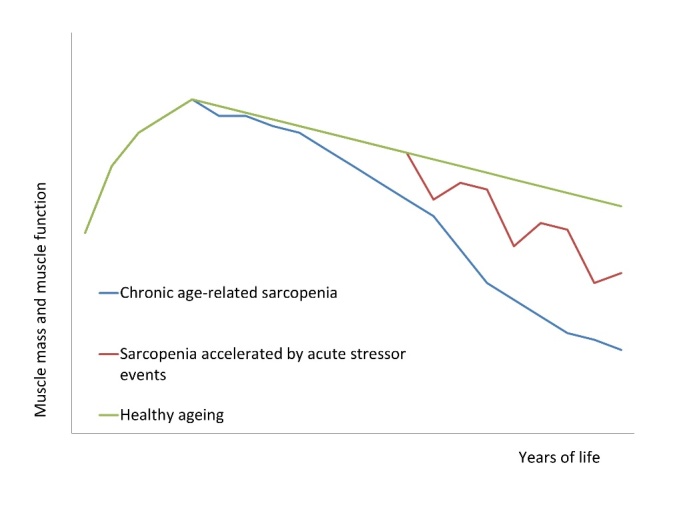
Proposed disease trajectories associated with sarcopenia This diagram demonstrates proposed trajectories associated with the development of sarcopenia over time. The green line demonstrates expected changes of muscle mass and function associated with healthy ageing; there may be some inevitable loss of muscle mass and function but not to such an extent as to cause detriment. The blue line demonstrates the development of chronic sarcopenia over time. The red line demonstrates our proposed model of how episodes of acute sarcopenia can potentially lead to the development of chronic sarcopenia over time.

As described, chronic age-related sarcopenia is associated with progressive decline over time secondary to an accumulation of insults [1]. We hypothesise that acute sarcopenia secondary to hospitalisation is associated with an acute rapid decline in muscle mass and muscle function. This may be partially recovered after discharge from hospital and recovery from illness, but may not return to pre-illness baseline [15]. This may lead to more rapid decline in muscle mass and function, resulting in chronic sarcopenia (see Fig. 2).

**Figure 3.**
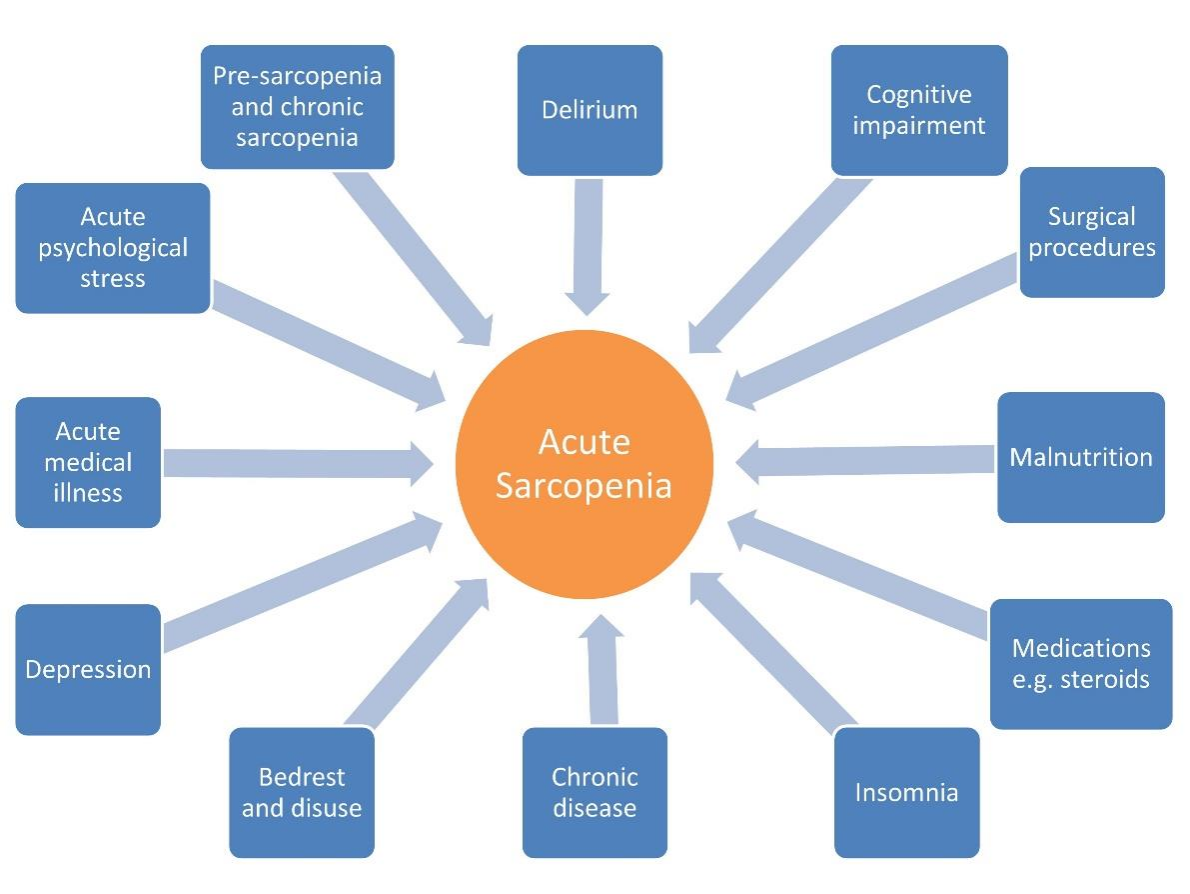
Potential effectors of "acute sarcopenia" secondary to hospitalisation There are likely to be multiple potential factors that can increase an individual’s likelihood of developing acute sarcopenia. Some of these are person-specific factors that lead to an increased predisposition to developing this condition; others relate to the stressor event itself. Many of these effectors are potentially amenable to prevention or intervention.

Identifying risk factors for acute sarcopenia may be important in preventing these long-term sequelae. Risk factors are likely to include, but not be confined to conditions that lead to endocrine dysregulation, increased inflammatory state and/or result in reductions in mobility; delirium [96], sleep disturbance [97], chronic cognitive impairment [98] and acute and chronic psychological stress [99], along with malnutrition (reduced protein intake) [100] are potential ameliorators (see Fig. 3).

However, long-term data relating to outcomes following episodes of acute loss of muscle mass and function are currently lacking. There is some evidence that reduced signalling through anabolic pathways is of benefit with regards to reducing cancer risk and increasing longevity. Clearly, during acute illness upregulation of catabolic pathways helps to produce energy to overcome illness. Loss of muscle mass and function may be a trade-off of this. Further research is needed in the form of observational studies and subsequent translational studies to assess potential targets and long-term risk from treatments.

### Acute sarcopenia: potential prevention strategies and treatments

#### Physical activity interventions

During hospital admission bed rest should be minimalised where possible [101]. This has wider health benefits beyond effect on muscle function, as bed rest is associated with increased risk of pressure ulcers [102], venous thromboembolism [103], pneumonia [104] and constipation [105]. Although the association between bed rest and adverse outcomes has been reported for several decades [106, 107], hospital inpatients often remain reclined to bed in clinical practice [101]. This can partially be explained by staff reluctance to allow early mobilisation due to an increased risk of falls leading to traumatic brain injury, fracture or other injury [108]. This may compound the problem, as declines in muscle mass and function lead to increased risk of falls [5].

There is increasing evidence that early mobilisation and intensive physical activity regimes can help to improve outcomes. A recently published trial involving 92 non-delirious older medical inpatients demonstrated increased mobility at one month follow-up in those randomised to an inpatient mobility program compared to those who received usual care; no differences in ADL performance were reported [109]. A previous systematic review of exercise interventions for hospitalised older adults also found that the impact on ADL performance was unclear, but reported a small but important reduction in length of hospital stay and overall costs [110]. In the case of elective admissions, prehabilitation may be considered in the form of increased physical activity prior to admission. A randomised control trial of prehabilitation vs. rehabilitation in patients undergoing colorectal surgery demonstrated similar complication rates between the two groups but a significantly improved 6 minute walking test (6MWT) in the prehabilitation group at eight week follow-up [111].

Resistance exercise training in combination with moderate aerobic exercise have been shown to be beneficial in improving muscle mass and function in chronic sarcopenia [112]. Resistance exercise training is likely to be beneficial in the prevention and treatment of acute sarcopenia, in combination with aerobic exercise, however there are likely to be limitations in the extent that this can be tolerated during the acute illness. The optimum duration and intensity of physical activity to treat or prevent acute sarcopenia is currently unknown and is an important area for future research.

#### Nutritional interventions

Nutritional interventions have been trialled in chronic sarcopenia and there is increasing evidence that older adults have higher protein requirements than younger adults, with further increased requirements during acute illness [113]. Leucine intake is particularly important [114]. Only 5% leucine is metabolised to β-hydroxy-β-methylbutyrate (HMB), thus direct administration of HMB may provide a more efficient alternative [115]. HMB administration prevents loss of lean leg mass in healthy older adults during bed rest [116] and has recently been demonstrated to reduce post-discharge mortality in a hospitalised population [117]. Protein supplementation can be implemented in the acute care setting alongside exercise interventions or alone, where early mobilisation is not possible [118].

There are clear health benefits for promotion of physical activity and nutritional optimisation. However, further research is needed to clarify appropriate dosages, duration, intensity and timing that provide economic as well as individual benefits.

#### Neuromuscular Electrical Stimulation (NMES)

Neuromuscular Electrical Stimulation (NMES) involves the application of electrical current to stimulate muscular contraction and has been trialled in situations where mobilisation is not possible, such as in the ICU setting. A recent randomised controlled trial involving critically ill patients following cardiothoracic surgery demonstrated no effect of NMES applied bilaterally to quadriceps on muscle layer thickness, although patients regained muscle strength faster than the control group. The mean age in the NMES group was 63.3, compared to 69.7 in the control group [89]. Conversely, a study using NMES applied unilaterally significantly prevented reductions in muscle fibre cross-sectional area compared to biopsies taken from the control quadriceps. The mean age of patients in this study was 70 [119]. NMES is, therefore, a potential strategy to prevent targeted muscle atrophy and loss of muscle strength, but is less feasible at preventing loss of total skeletal muscle.

#### Pharmacological therapies

Novel pharmacological agents for treatment of sarcopenia include calorie restriction mimetics (CRMs) and exercise mimetics (EMs); these are phytochemicals such as resveratrol with antioxidant and regenerative properties that partially mimic the molecular pathways leading to the favourable effects of calorie restriction and physical exercise [120]. If proven beneficial, these may also provide benefit in the acute care setting in preventing acute loss of muscle mass and function.

Recombinant growth hormone administration has previously been trialled with conflicting results. Growth hormone supplementation can increase skeletal muscle mass, but this is rarely accompanied by improved muscle strength and side-effects of this treatment are prevalent in older adults [121]. Testosterone supplementation to older men can increase muscle mass and muscle function [122, 123] but is associated with significant increased adverse events including increased rates of prostate cancer and measured Prostate Specific Antigen (PSA) levels [124]. Trials of supplementation with the androgen DHEA are extensive and results are contradictory. A recent systematic review reported that 5 out of 7 eligible studies showed improvement in single aspects of muscle function, such as grip strength or leg extensor strength, though only 1 showed improvement in a composite score of muscle strength [125]. Benefits of DHEA for sarcopenic adults remain to be established.

## Conclusions

Acute sarcopenia secondary to hospital admission is hypothesised to be a related but distinct condition from chronic age-related primary sarcopenia. Acute illness or surgery lead to an acutely heightened inflammatory burden, which, coupled with reduced physical activity and muscle disuse, can lead to a reduction in muscle mass and function. The natural history of this condition needs further evaluation, including factors that affect rate and extent of development of this condition and long-term outcomes.

It is unclear to what extent acute sarcopenia differs from chronic age-related sarcopenia in terms of biological and structural changes, physical phenotyping and long-term outcomes. Further research is needed on this subject, initially to demonstrate the association between hospitalisation, acute illness or surgery and sarcopenia and, subsequently, to examine long-term outcomes and the effect of early interventions including physical therapy, nutritional supplementation or using novel therapeutic targets. In-vivo physiology and muscle biopsy studies will be vital in assessing mechanisms driving acute sarcopenia.

Acute sarcopenia should be considered as a separate entity to chronic age-related sarcopenia, much in the same way that acute kidney injury is considered a separate disease to chronic kidney disease. The same criteria may be met for the two conditions, but it is the timing and natural history of the conditions that differ. However, considering the analogy above, there are likely to be overlap between the two conditions, with chronic sarcopenia being a possible risk factor for acute sarcopenia and the development of acute sarcopenia potentially increasing the long-term risk of chronic sarcopenia.

The presence of low muscle mass and function during acute illness is associated with increased health economic costs in terms of increased length of hospital stay, rehabilitation costs and the need for institutional care or social care on discharge. Preventing acute sarcopenia will have wider economic benefits as well as individual benefit to patients.

Initial research should determine the feasibility of conducting research on this condition in the acute care setting. The aim is that observational studies should be conducted within the next five years to include evaluation of histological changes and biological mechanisms. Focus on specific postulated risk factors, such as delirium will guide future treatment. Interventional studies involving nutritional, physical activity and other interventions should continue concurrently with assessment of general health and economic benefits. Prevention and early treatment of acute sarcopenia may help to prevent older adults from meeting criteria for sarcopenia in the long-term.
